# Time-Dependent Reliability Analysis of Reinforced Concrete Beams Subjected to Uniform and Pitting Corrosion and Brittle Fracture

**DOI:** 10.3390/ma14081820

**Published:** 2021-04-07

**Authors:** Mohamed El Amine Ben Seghier, Behrooz Keshtegar, Hussam Mahmoud

**Affiliations:** 1 Division of Computational Mathematics and Engineering, Institute for Computational Science, Ton Duc Thang University, Ho Chi Minh City 700000, Vietnam; 2 Faculty of Civil Engineering, Ton Duc Thang University, Ho Chi Minh City 700000, Vietnam; 3 Department of Civil Engineering, Faculty of Engineering, University of Zabol, Zabol P.B. 9861335856, Iran; bkeshtegar@uoz.ac.ir; 4 Department of Civil and Environmental Engineering, Colorado State University, Fort Collins, CO 80523, USA; Hussam.Mahmoud@colostate.edu

**Keywords:** reinforced concrete beams, pitting corrosion, general corrosion, reliability analysis, three-term conjugate FORM

## Abstract

Reinforced concrete (RC) beams are basic elements used in the construction of various structures and infrastructural systems. When exposed to harsh environmental conditions, the integrity of RC beams could be compromised as a result of various deterioration mechanisms. One of the most common deterioration mechanisms is the formation of different types of corrosion in the steel reinforcements of the beams, which could impact the overall reliability of the beam. Existing classical reliability analysis methods have shown unstable results when used for the assessment of highly nonlinear problems, such as corroded RC beams. To that end, the main purpose of this paper is to explore the use of a structural reliability method for the multi-state assessment of corroded RC beams. To do so, an improved reliability method, namely the three-term conjugate map (TCM) based on the first order reliability method (FORM), is used. The application of the TCM method to identify the multi-state failure of RC beams is validated against various well-known structural reliability-based FORM formulations. The limit state function (LSF) for corroded RC beams is formulated in accordance with two corrosion types, namely uniform and pitting corrosion, and with consideration of brittle fracture due to the pit-to-crack transition probability. The time-dependent reliability analyses conducted in this study are also used to assess the influence of various parameters on the resulting failure probability of the corroded beams. The results show that the nominal bar diameter, corrosion initiation rate, and the external loads have an important influence on the safety of these structures. In addition, the proposed method is shown to outperform other reliability-based FORM formulations in predicting the level of reliability in RC beams.

## 1. Introduction

RC beams are widely used in the construction of many structures and infrastructure such as buildings and bridges [1,2,3]. However, as infrastructures age, the strength of RC beams could be compromised, particularly in a corrosive environment [4,5,6]. To avoid any risk of damages or failure, deteriorated beams can be replaced or rehabilitated [7,8]. Funding limitations, however, could hamper the ability to repair or replace the beams when needed [9,10,11]. Therefore, it is of paramount importance to quantify the service life and reliability of RC beams that are subjected to aggressive environmental conditions which could lead to corrosion of the reinforcing steel. Corrosion is one of the main issues for structures made of steel that can have a high risk of failure according to several studies [12,13,14,15,16,17]. The development of an appropriate formulation to capture the behavior of RC beams subjected to different corrosion forms with an accurate structural reliability framework will enable owners and field engineers to make more risk-informed decisions to increase the service life of the structure by performing the required maintenance actions.

Considering the complex behavior of RC beams under corrosion effects, the accurate estimation of the failure probability (*P_f_*) is required [18]. Among the most useful approaches for quantifying the safety levels of the structures is using structural reliability analysis (SRA), which can be performed using the so-called performance function or limit state function (LSF) [19]. Performance function or LSFs are explicit design equations that include various basic variables to describe the failure mode or the structural behavior. Accounting for uncertainties in these variables can be realized by assigning specific statistical distributions to these functions to represent their randomness [20]. Thereafter, different SRA approaches can be applied to predict the failure probability (*P_f_*) under the pre-selected condition of the structure. Various techniques have been developed over the years to conduct safety assessments of different structures, which can be grouped into simulation- and analytical-based approaches [21,22,23]. The Monte Carlo simulation (MCS) is a popular simulation technique that used to solve many problems in different engineering fields [24,25]. However, MCSs can be computationally expensive, especially when the LSF is obtained from a finite element model (FEM) [26]. To overcome the MCS problem, reduction techniques are employed to reduce the number of simulations, such as the importance sampling technique (IS), subset simulation (SS), line sampling (LS) and directional sampling (DS) techniques [27,28]. Even though, these reduction techniques were found to be efficient, their accuracies are moderate for LSFs that are highly nonlinear or characterized by large dimensions. Accurate convergence of these techniques also requires prior information of the failure regions.

In contrast, analytical approaches, including the first and second order reliability method (FORM and SORM), only require an estimate of the most probable point of failure on the LSF surface (MPP); hence, they are computationally more efficient and accurate [29]. Despite their attractiveness, various drawbacks can be associated with the use of FORM and SORM. For example, the FORM can provide unstable estimations related to problems with variables that are characterized by non-normal distributions, similar to those of RC beams. Modified versions of the FORM approach have been developed over the years to enhance its ability to accurately estimate the MPP to fail on the surface of the performance function [30,31]. These improvements include two types of enhancements: steepest descent and conjugate search directions [32]. Steepest descent techniques are formulated based on a step size with values inferior to 1, including, as an example, the relaxed HL-RF (RHL-RF), the finite-step length (FSL) method, the directional STM (DSTM), and the adjusted finite step length method [33]. These approaches are better than the classic algorithm, though computationally inefficient for moderately complex LSFs. On the other hand, conjugate search direction techniques, which use a finite-step size, include methods such as the conjugate chaos control (CCC) method, the relaxed conjugate reliability (RCR) approach, and the adaptive conjugate method [32]. These approaches have proven to be more efficient for the SRA than the first group; however, the main effort is to accurately formulate the conjugate scalar factor in FORM. Therefore, the FORM-based conjugate search direction may be a reliable approach to solve the estimation of the safety levels of RC beams using the SRA with a new formulation of the conjugate scalar factor.

To overcome these drawbacks, the development of an efficient, reliable technique that is able to provide accurate results is critical for reliability analyses of RC beams. Besides, accurate estimation of the reliability requires robust formulation of the LSF pertaining to the corrosion formation in the beams. Consequently, an improved reliability method based on FORM is introduced in this study for the reliability analysis of RC beams, where the LSF is formulated under two types of corrosions: uniform and pitting corrosion. Moreover, various investigations are carried out to evaluate the effect of the basic random variables selected in the analysis on the outcome of the results. The paper is structured as follows: Section 2 describes the problem formulation and the limit state of the reinforced concrete beams development under the two corrosion forms; Section 3 introduces the proposed reliability framework, while the numerical validation of the proposed method abilities is investigated; Section 4 reports the probabilistic forms of the design variables for the case study; Section 5 represents the results of the performed reliability analysis with detailed discussions; and the conclusions, limitations, and recommendations for future work are presented in Section 6.

## 2. Mathematical Formulation of the Corroded Reinforced Concrete Beam

The development of an appropriate model for the LSF that describes the failure mode of reinforced concrete beams under two types of corrosion forms (uniform and pitting corrosion) is addressed in this section. These two corrosion forms can affect the reinforced concrete beams in two successive processes, beginning with corrosion initiation prior to corrosion propagation, in which these two processes can be explained as follows:

(1) First, the time to corrosion initiation starts with corrosion activation on the steel bars, in which initiation begins when the received corrosive ions contact the bar’s surface.
(2) Second, the time of the corrosion propagation; in this period, the corrosion defects/cracks are propagated and the cross-sectional areas of the reinforcing steel bars decreases, leading to bond strength reduction and subsequent structural performance degradation.

### 2.1. Limit State Function Formulation

**Figure 1:**
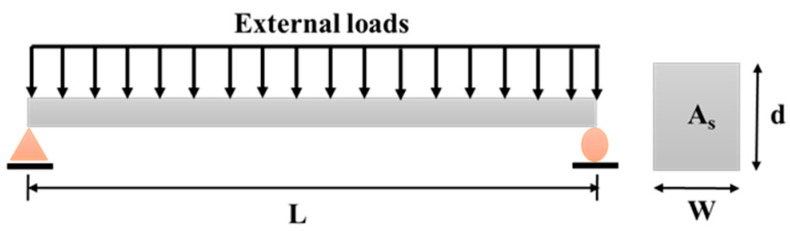
Reinforced concrete beam under a distributed load.

According to Stewart, the LSF of a typical simply supported corroded RC beam subjected to distributed loads, as illustrated in Figure 1, can be defined under the flexural failure mode by the maximum bending moment at mid span [34,35,36]. It should be mentioned that the beam with a rectangular cross-section is usually reinforced with a specific number of bars, each with an equal diameter. Thus, the LSF in this study can be given as follows [37,38]:

(1a) $$G\left(M\right)=\eta A\left(t\right)f_{y}\left(t\right)\left(d-K\frac{A_{s}\left(t\right)f_{y}\left(t\right)}{bf_{c}}\right)-\lambda M_{u}$$

(1b) $$f_{y}\left(t\right)=\left(1-\alpha \frac{A_{s}-A_{s}\left(t\right)}{A_{s}}\right)f_{y}$$

where *η* and α are empirical coefficients representing the model uncertainty related to the flexural and tension resistance of the bars, respectively; *A_s_* is the cross-sectional area of the reinforcing steel mm^2^; *t* refers to the time, *f_c_* and *f_y_* represent the concrete compressive strength and the steel yield stress in MPa, respectively; *d* denotes the effective height in mm; *b* is the section width in mm; *K* is the resistance ratio; *λ* is the load coefficient; and *M_u_* is the applied moment (kN-m). Noting that the cross-section areas are highly dependent on the corrosion process forms (i.e., general and pitting corrosion), these two forms can be mathematically formulated for the reinforced bars as explained in the following sections.

### 2.2. Uniform Corrosion Model

The mathematical model for the time-dependent loss of the bar cross-section subjected to uniform corrosion can be expressed using Equation (2) as follows [39,40]:

(2) $$A_{s}\left(t\right)=\frac{\pi {\left(D_{0}-2P_{av}\right)}^{2}}{4}$$

where *D*_0_ represents the nominal bar diameter (mm); *t* is the elapsed time since corrosion initiation (year); and *P_av_* denotes the progression of the average pit depth over time (mm/year), which can be calculated using:

*P*_*av*_ = 0.0116 *i*_*corr*_ (*t*) *t* (3)

In Equation (3), *i_corr_* (*t*) represents the rate of corrosion (μA/cm^2^), which can be computed as:

*i_corr_*(*t*) = 0.85 *i_corr_*(1) *t*^−0.29^ (4)

where *i_corr_*(1) refers to as the corrosion rate (μA/cm^2^) at the beginning of corrosion propagation, which can be calculated using:

(5) $$i_{corr}\left(1\right)=\frac{37.8{\left(1-wc\right)}^{-1.64}}{c}$$

In Equation (5), *C* denotes the cover of the concrete (cm); *wc* is the water–cement ratio of the concrete, which can be calculated using Equation (6) as a function of the concrete compressive strength (i.e., the Bolomey’s formula) [41]:

(6) $$wc=\frac{27}{f_{cy}^{\prime }+13.5}\;\;\;\;,\;f_{cy}^{\prime }=f_{c}^{\prime }+7.4\;\mathrm{MPa}$$

### 2.3. Pitting Corrosion Model

The mathematical model for the time-dependent loss of the bar cross-section due to pitting corrosion can be expressed using Equation (7) as follows [42,43]:

(7) $$A_{p}(t)=\{\begin{array}{cc} A_{1}+A_{2} & P(t)\leq \frac{D_{0}}{\sqrt[{}]{2}}\\ A_{s}-A_{1}+A_{2} & \frac{D_{0}}{\sqrt[{}]{2}}\leq P(t)\leq D_{0}\\ A_{s} & P(t)>D_{0} \end{array}$$

where *P(t)* denotes the time-dependent maximum penetration of the pitting corrosion, which can be estimated using Equation (8); *A_s_* is the free-corrosion cross-section area; and *A_1_* and *A_2_* are the corroded cross-sections calculated based on Equation (9):

*P*(*t*) = 0.0116 *i*_*corr*_(t) *Rt* (8)

(9) $$\begin{array}{c} A_{s}=\frac{\pi D_{0}^{2}}{4}\\ A_{1}=0.5\left[\theta_{1}{\left(\frac{D_{0}}{2}\right)}^{2}-b\left|\frac{D_{0}}{2}-\frac{P{\left(t\right)}^{2}}{D_{0}}\right|\right]\\ A_{2}=0.5\left[\theta_{2}P{\left(t\right)}^{2}-b\frac{P{\left(t\right)}^{2}}{D_{0}}\right]\\ b=2P\left(t\right)\sqrt{1-{\left(\frac{P\left(t\right)}{D_{0}}\right)}^{2}}\;,\;\theta_{1}=2\arcsin\left(\frac{b}{D_{0}}\right),\;\theta_{2}=2\arcsin\left(\frac{b}{2P\left(t\right)}\right)\; \end{array}$$

In Equation (9), *R* represents the ratio between the pitting corrosion and the average depth as $R=\frac{P\left(t\right)}{P_{av}}$, and is called the pitting factor. Thus, the resulting cross-section area of the steel bar under pitting corrosion can be calculated as follows:

(10) $$A_{s}\left(t\right)=A_{s}-A_{p}\left(t\right)$$

Unlike uniform corrosion, the pits from corrosion can evolve into fatigue cracks, causing complete fracture of the rebar. According to a previous study by Masoud et al. [44], the fatigue life of RC beams can be reduced significantly due to pit corrosion. Thus, the fatigue life due to cracks originating from pits should be investigated. To determine the number of cycles to failure (*N*) for a cracked element, linear elastic fracture mechanics (LEFM) can be employed. The Paris law can be used to define the relationship between the rate of crack propagation (*da*/*dN*) as a function of the stress intensity factor range (∆*K*) as follows:

(11) $$\frac{da}{dN}=C∆K^{m}$$

where *N* denotes the number of fatigue cycles with stable crack growth; while *C* and *m* are material constants. The expression of the stress intensity factor for mode *I* (opening mode) can be given as function of the nominal stress (*σ*), the crack size (*a*), and the correction factor (*F*) as follows [45]:

(12) $$K_{I}=F\sigma \sqrt{\pi a}$$

In Equation (12), *F* can be computed for a circular steel reinforcing bar with pit corrosion as follows [46]:

(13) $$F=\frac{\frac{1.84}{\pi }{\left[\frac{\tan\left(\frac{\pi a}{4r}\right)}{\frac{\pi a}{4r}}\right]}^{0.5}}{\cos\left(\frac{\pi a}{4r}\right)}\left\{0.752+2.02\left(\frac{\pi a}{4r}\right)+0.37{\left[1-\sin\left(\frac{\pi a}{4r}\right)\right]}^{3}\right\}$$

In case the value of the stress intensity factor ($K_{I}$) reaches the fracture toughness (*K_IC_*), which is associated with the critical crack value attained, fracturing will occur. The limit state function can be modeled as the difference between the fracture toughness and the stress intensity factor (Equation (14)), where *a* is replaced by *P*(*t*) using Equation (8), and the value of *K_IC_* can be obtained from experimental tests by first determining the Charpy V-notch values [47].

(14) $$LSF\left(X,t\right)=K_{IC}-K_{I}$$

Equations (2)–(14) are utilized and integrated into the LSF to calculate the meta-loss in the cross-sectional areas due to uniform and pitting corrosion and to estimate the probability of failure caused by cracks originating from pits for the RC beam.

## 3. Proposed Reliability Approach

As stated before, two formulation types based on the normalized sensitivity vector are used to enhance the performance of the analytical FROM approach, including the steepest descent and conjugate sensitivity vector-based improvements [48]. The HL-RF is the most well-used steepest descent technique for structural reliability. However, this technique shows inaccurate convergence or periodic solutions for highly nonlinear problems [49]. To overcome these drawbacks, extended versions of the FORM-based steepest decent sensitivity vector have been developed, including the chaos control (CC) [50,51], the relaxed HL-RF (RHL-RF) [33], the finite-step length (FSL) [52], the adjusted finite step length (AFSL) [53], and the non-negative constraint method (NCM) [54] approaches. These formulas have enhanced the original FORM abilities and robustness; however, their efficiency is limited to moderately nonlinear limit state functions. On the other hand, the conjugate sensitivity vector-based improvements have shown more promising results regarding the efficiency performance of the FORM method for moderate and highly nonlinear complex limit state functions. This includes using the adaptive conjugate scheme (ACS) [48], the conjugate chaos method (CC) [55], the limited conjugate method (LCM) [56], and the relaxed conjugate approach (RCA) [57] in comparison with the first category (i.e., steepest descent formulas such as CC and FSL) for solving various structural reliability analysis problems.

Consequently, as the FORM-based conjugate sensitivity vectors are efficient formulations for estimating the failure probability, Keshtegar and Zhu developed a novel three-term conjugate map for structural reliability analyses with robust and efficient formulation [58]. This method can be used for applicable engineering problems for the evaluation of the safety levels. Thus, in this study, the conjugate sensitivity vector is developed for the reliability analysis of corroded RC beams. Specifically, the three-term conjugate finite-step length is introduced for the reliability analysis of RC beams under two types of corrosion with large uncertainties. The three-term conjugate sensitivity vector is formulated using two factors for adapting the normalized vector in FORM to adjust the gradient and conjugate gradient vectors.

**Figure 2:**
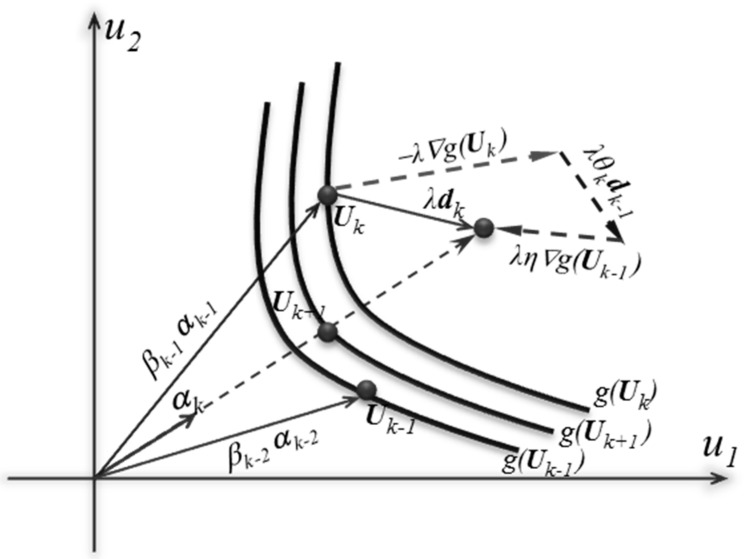
Illustration of the proposed framework process using the three-term conjugate map (TCM) algorithm, modified from [58].

Figure 2 illustrates the principle of FORM in two-dimensional standard normal space (*U*). Figure 2 shows the main steps for the estimation of the value of the reliability index, *β*, using the proposed TCM method. Thus, to determine the new point ($U_{k+1}$) in the FORM technique, the conjugate discrete map is used as follows:

(15) $$U_{k+1}=\beta_{k}\alpha_{k}\;\beta_{k}=\frac{\nabla^{T}g\left(U_{k}\right)U_{k}-g\left(U_{k}\right)}{\nabla^{T}g\left(U_{k}\right)\alpha_{k}}$$

where $\beta_{k}$, $\nabla g\left(U_{k}\right)$, and $\;g\left(U_{k}\right)$ denote the reliability index, the performance function gradient vector, and the LSF at point ***U_k_***, respectively. ***α_k_*** represents the normalized conjugate sensitivity vector and is calculated using the following equation:

(16) $$\alpha_{k}=\frac{U_{k}+\lambda d_{k}}{||U_{k}+\lambda d_{k}||}$$

where λ denotes the finite-step length  λ≥0 and ***d_k_*** represent the conjugate vector that can be computed using the following formulas:

(17) $$\begin{array}{c} d_{k}=-\nabla g\left(U_{k}\right)+\theta_{k}d_{k-1}+\eta_{k}\nabla g\left(U_{k-1}\right)\\ \eta_{k}-min\left\{0.9\frac{{||\nabla g(U_{k})||}^{2}}{{||\nabla g(U_{k-1})||}^{2}},\frac{{||\nabla g(U_{k})||}^{2}}{{||d_{k-1}||}_{2}}\right\}\\ \theta_{k}=-\;\frac{{\nabla g||g(U_{k})||}^{2}}{{||\nabla g(U_{k-1}||)}^{2}} \end{array}$$

In Equation (17), $\theta_{k}$ and $\eta_{k}$ are the two conjugate factors that are used to adjust the new conjugate vector.

Figure 2 schematically illustrates the iteration process of the FORM based on the proposed three-term conjugate sensitivity vector, where three points $g\left(U_{k-1}\right)$, $g\left(U_{k}\right)$ and $g\left(U_{k+1}\right)$ are shown on the LSFs. From the drawn normalized sensitivity vector, the conjugate vector can control the FORM robustness and accuracy using two adjusting factors. The factors are used to combine the current gradient and the previous conjugate gradient vectors to achieve a more robust solution. Besides, $\alpha_{k}$ is not paralleled with the previous conjugate gradient vectors. Thus, this can reduce the risk of oscillating solutions for highly nonlinear problems.

Using the above relations of the classic FORM in this current work, the three-term conjugate sensitivity vector is used to compute the reliability index through the following steps:

1. Establish the performance function g(U)=0 and specify the basic random variables of the problem.
2. Set *k* = 0, *d*_0_ = 0 and $\eta_{0}=0$ and specify the convergence limit criteria, ε ($\varepsilon =10^{-6}$ was adopted in this work).
3. Transfer the basic random variables from *X* space to *U* space.
4. Compute the values of $\nabla g\left(U_{k}\right)$ and $g\left(U_{k}\right)$.
5. Determine $\lambda_{0}=\frac{100}{||\nabla g\left(U_{|U=\mu }\right)||}$ at *k* = 0.
6. Compute the conjugate factors as $\theta_{k}=-{\nabla g||\left(U_{k}\right)||}^{2}/{||\nabla g\left(U_{k-1}\right)||}^{2}$ and $\eta_{k}-min\left\{0.9{||\nabla g\left(U_{k}\right)||}^{2}/{||\nabla g\left(U_{k-1}\right)||}^{2},{||\nabla g\left(U_{k}\right)||}^{2}/{||d_{k-1}||}^{2}\right\}$.
7. Compute $d_{k}$ using the three-term formulation as $d_{k}=-\nabla g\left(U_{k}\right)+\theta_{k}d_{k-1}+\eta_{k}\nabla g\left(U_{k-1}\right)$.
8. Compute the normalized conjugate sensitivity vector, i.e., $\alpha_{k}$.
9. Determine the results for the new iterations as $\beta_{k}=\frac{\nabla^{T}g\left(U_{k}\right)U_{k}-g\left(U_{k}\right)}{\nabla^{T}g\left(U_{k}\right)\alpha_{k}}$ and $U_{k+1}=\beta_{k}\alpha_{k}$.
10. If $||U_{k}-U_{k-1}||\geq \varepsilon$, then *k* = *k* + 1 and go to Step 3; else, stop and print $X^{*}=X_{k+1}$, $U^{*}=U_{k+1}$, $\beta_{k}$ and $P_{f}\approx \Phi \left(\beta_{k}\right)$.

## 4. Probabilistic Form of the Basic Random Variables for the Corroded RC Beam

After examining the applicability and the performance of the proposed reliability analysis method, namely TCM (see Section 3), and the formulation of the LSFs of the reinforced concrete beam that was subjected to two different types of corrosion form (uniform and pitting corrosion; see Section 2), the reliability analysis was performed on a case study in which the influence of various variables, such as the bar diameters, the applied load, the corrosion initiation, and the compressive concrete resistance, was investigated. The reliability analysis was carried out based on the limit states function using Equation (1), while the effect of corrosion types on the RC beam was modeled using Equations (2)–(14). The random basic variables involved in the reliability analysis of the reinforced concrete beam are reported in Table 1. The associated mean, coefficient of variation (CoV), and the attributed distribution were extracted based on the previous studies in this field [59,60,61,62,63,64].

## 5. Application, Results and Discussion

**Figure 3:**
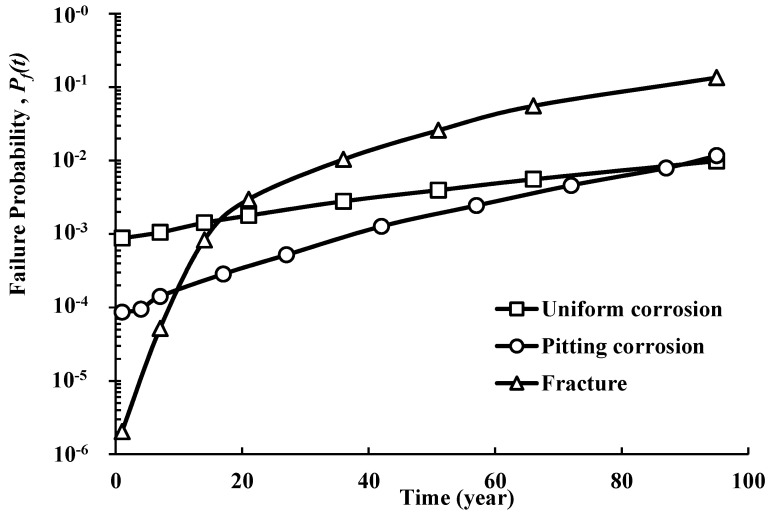
Time-dependent failure probability of steel bars subjected to general and pitting corrosion.

Using the proposed TCM approach, the structural reliability analysis of a reinforced concrete beam is carried out under both forms of corrosion (uniform and pitting corrosion), including brittle failure mode using the formulated LSFs in Section 2. The results of the reliability analysis were described in term of failure probability (i.e., reliability index). Moreover, the influence of several parameters on the reliability of the reinforced concrete beam was investigated, including the nominal diameter of the bars, *D*_0_ (mm), the corrosion rate at the beginning of corrosion propagation, *i_corr_*(1) (μA/cm^2^), the concrete compressive resistance, *f_c_* (MPa), and the applied moment (i.e., external load), *M_u_* (kN-m). A comparison of the time-dependent failure probability due to uniform and pitting corrosion forms and fracture mode is illustrated in Figure 3. It should be mentioned that the selected value of the nominal diameter of the bars, *D*_0_, was 18 mm, while the other variables were selected as indicated in Table 1. According to the obtained reliability results, the probabilities of failure for the RC beam due to uniform corrosion are higher than that of pitting corrosion. This conclusion can be attributed to several reasons, including, for example, the higher likelihood of uniform corrosion to occur on the steel surface than pitting corrosion. From a mathematical point of view, this relates to the developed models in this field, where more restrictions and a classical fitting technique were used to develop the above models and correlations. However, the occurrence probability of fracture due to the transition from a pit to crack defect shows a different risk of pitting corrosion on the RC beam, where for small pits/cracks, the probability of failure due to fracture is small compared to uniform corrosion, but with the progression of time, the fracture failure probabilities increase rapidly. This indicates that despite the lower failure probabilities due to pitting corrosion, failure probabilities can substantially increase due to the fracture. Overall, Figure 3 clearly shows that increasing both pitting and uniform corrosion with time will lead to large reduction in the strength of the RC beam. As an example, for a failure probability of 0.004, the beam can reach a service life time of 53 years in the case of uniform corrosion, 70 years in the case of pitting corrosion, and only 20 years to complete fracture due to cracks propagating from pits.

### 5.1. Effect of the Steel Bars Diameters (D_0_, mm)

**Figure 4:**
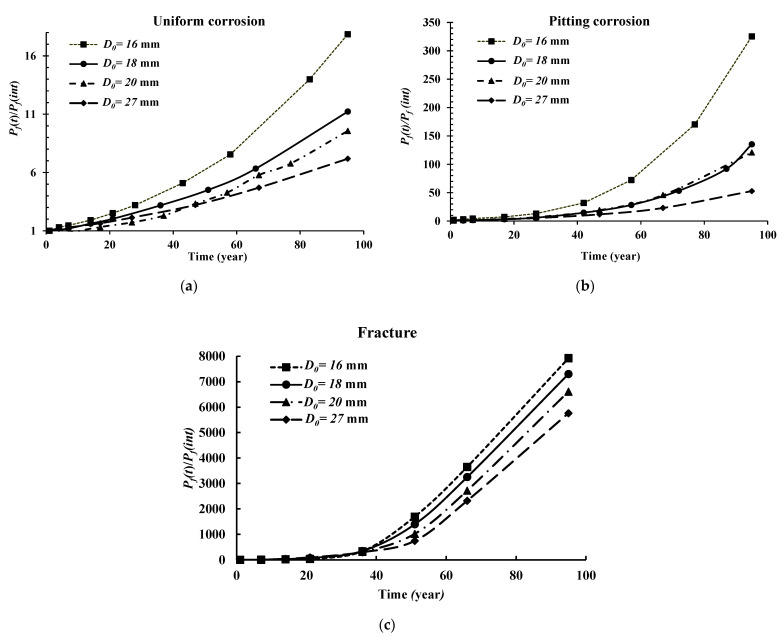
Influence of the nominal diameter of the bar (*D*_0_) on the reliability analysis results for the (**a**) uniform corrosion form, (**b**) pitting corrosion form, and (**c**) fracture failure mode.

To address the influence of the nominal diameter, *D*_0_, of the steel bars on the failure probability (*P_f_*) of the reinforced beam structure, four different diameters were used as the mean values: 16 mm, 18 mm, 20 mm, and 27 mm. The time-dependent reliability analysis results are shown in Figure 4a–c for the uniform, pitting, and fracture-based failure modes, respectively. It should be noted that these results are illustrated in terms of the ratio of the time-dependent failure probability with corrosion (i.e., uniform, pitting or fracture) to the failure probability at the beginning of corrosion initiation (*t* = 1). Therefore, the higher this ratio, the higher the impact caused by the mode of failure. As expected, an increase in the nominal diameter of the bars resulted in a reduction in the failure probability (i.e., $P_{f}^{D_{0}=27\;\mathrm{mm}}\;<\;P_{f}^{D_{0}=20\;\mathrm{mm}}<\;P_{f}^{D_{0}=18\;\mathrm{mm}}<\;P_{f}^{D_{0}=16\;\mathrm{mm}}$). In addition, after 50 years of service life, the influence of the diameter is amplified. Another observation is that the values of the ratio $P_{f}^{Corrosion}$/$P_{f}^{Intiation}$ for the case of pitting corrosion are much higher than for uniform corrosion, while the probability of failure due to fracture, caused by pitting corrosion, is found to be the highest.

**Figure 5:**
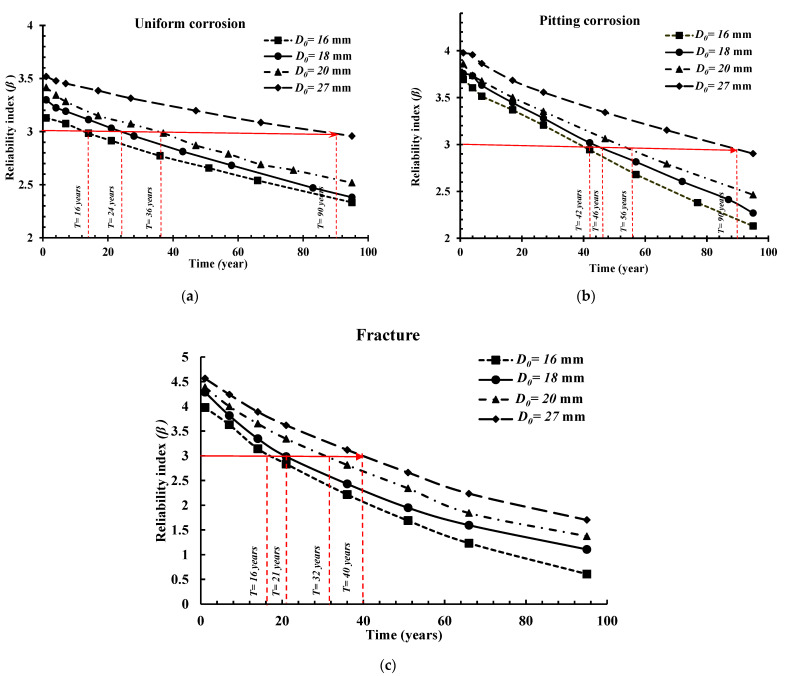
Influence of the nominal diameter of the bar (*D*_0_) on the reliability index results for the (**a**) uniform corrosion form, (**b**) pitting corrosion form, and (**c**) fracture failure mode.

Figure 5 illustrates a comparative study between the permitted predictable service life of the RC beam under the studied corrosion forms (uniform and pitting) and the fracture failure mode based on a safety threshold equal to *β* = 3 in terms of reliability index. In addition, the four cases based on the differing nominal diameters (*D*_0_) were considered. Therefore, using this threshold, the predictable allowed service life of the RC beam under uniform corrosion for a *D*_0_ of 16 mm, 18 mm, 20 mm and 27 mm are 16, 24, 36 and 90 years, respectively. For the case of pitting corrosion, the results are 42, 46, 56 and 90 years in the same respect. The lowest predicted service life is captured in the fracture failure mode due to pit-to-crack failure as 16, 21, 32 and 40 years using 16 mm, 18 mm, 20 mm and 27 mm, respectively. After achieving the predicted service life, maintenance and repair actions should be taken to prevent failures and the associated consequences.

### 5.2. Effect of the Corrosion Imitation Time (icorr(1), μA/cm^2^)

**Figure 6:**
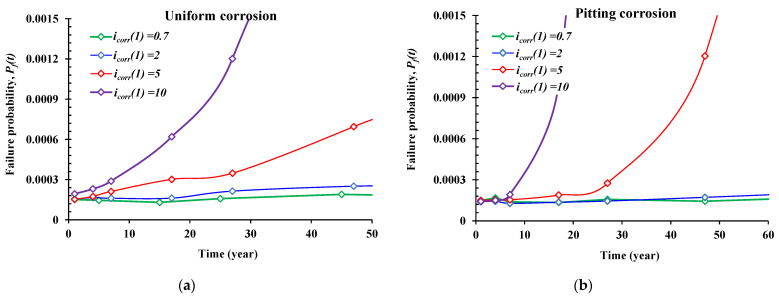
Influence of the corrosion rate at the beginning of propagation (*icorr*(1)) on the reliability results. (**a**) Uniform corrosion form, (**b**) pitting corrosion form.

Similar to the previous section, the influence of the initiation corrosion rate (*i_corr_*(1)) was also investigated. Figure 6 depicts the time-dependent reliability results versus four cases of *i_corr_*(1): 0.7 μA/cm^2^, 2 μA/cm^2^, 5 μA/cm^2^*,* and 10 μA/cm^2^, respectively. According to the results, the initiation corrosion rate (*i_corr_*(1)) has an important influence on the time-dependent failure probability of the reinforced concrete beam for the cases of *i_corr_*(1) = 5 and 10 μA/cm^2^, especially after 7 years of service. Therefore, the higher the value of (*i_corr_*(1)), the higher the probability of failure. Pitting corrosion affects the failure probability more than the case of uniform corrosion. For the cases of *i_corr_*(1) < 2 μA/cm^2^, it seems that this parameter has less influence on the reliability results, noting that this parameter is highly influenced by the surrounding environment conditions of the RC beam. Thus, it is very important to quantify the related condition of the structures for the accurate prediction of the safety levels.

### 5.3. Effect of the Concrete Compressive Resistance (f_c_, MPa)

**Figure 7:**
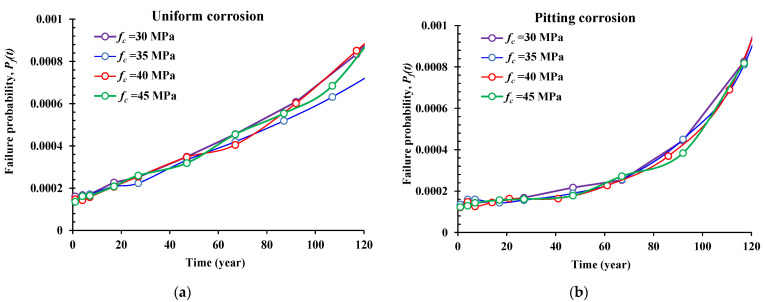
Influence of the concrete compressive resistance (*f_c_*) on the reliability results. (**a**) Uniform corrosion form, (**b**) pitting corrosion form.

Figure 7 illustrates the reliability results of the presented problem under four different cases of concrete compressive resistance (*f_c_*) in the range of 30 to 45 MPa for the two corrosion forms. Unlike the influence of the nominal diameter and the initiation corrosion rate, the compressive resistance of the concrete was found to have almost the same impact on the failure probability results for the four proposed values. However, it is shown that this parameter has an increasing linear influence with respect to time for the uniform corrosion form, and an almost exponential influence for the pitting corrosion form, especially after *T* > 60 years.

### 5.4. Effect of the Applied Moment (M_u_, KN-m)

**Figure 8:**
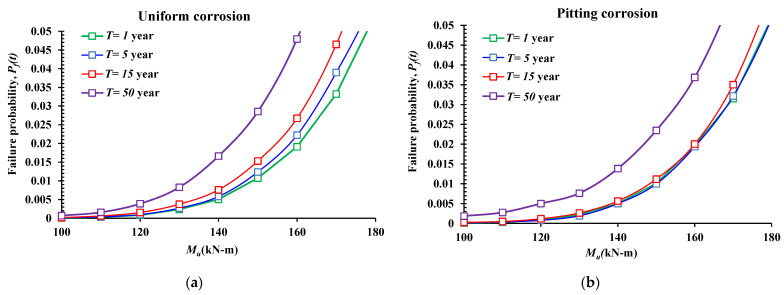
Influence of the external load (*M_u_*) on the reliability results. (**a**) Uniform corrosion form, (**b**) pitting corrosion form.

The final investigated effect was the external moment applied onto the RC beam subjected to uniform or pitting corrosion. Figure 8 illustrates the failure probabilities versus the applied moment within a range of 100 and 180 KN-m for different stages of service life for the beam at 1, 5, 15, and 50 years. As expected, the higher the applied load, the higher the failure probability for both corrosion forms, and for all the stages of the structure life. In addition, the corroded reinforced beam is more vulnerable when the service life reaches 50 years. It can also be observed that the influence of general corrosion, in accordance with the external moment, is uniform, where the older the structure, the higher the failure probability. However, for pitting corrosion, the results show that the effect was similar in accordance with age, which can be explained by the high risk to fail due to the concentrated penetration (pit) of the corrosion (unlike the general (uniform) corrosion on the bar’s surface).

## 6. Conclusions

To investigate the behavior of the safety levels of reinforced concrete beams that are subjected to uniform and pitting corrosion conditions in terms of failure probability, a new reliability method was utilized in this research. Thus, an improved version of the FORM method using a new conjugate sensitivity vector based on improvements to the three-term conjugate map was utilized. In addition, the flexural LSF was formulated based on previous research correlations and an empirical model to describe both the uniform and pitting corrosion forms. Moreover, the risk of the fracture due to pitting corrosion defects was formulated as the difference between the fracture toughness and the stress intensity factor.

The performance of the proposed TCM approach was validated against several structural reliability methods using complex and highly nonlinear examples. The obtained results indicated that the TCM method outperforms the others in terms of robustness, efficiency, and accuracy. The time-dependent reliability results showed that increasing the nominal diameter of the bars will decrease the failure probability, while it was found that the influence of uniform corrosion is more severe that pitting corrosion if the fracture mode is not considered. As an example, for *D*_0_ = 18 mm and a threshold of *β* = 3, the concrete beam structure achieves an allowed service life of 24 and 46 years for the cases of uniform and pitting corrosion, respectively, and only 21 years in the case of brittle fracture failure mode. Investigating the failure due to brittle fracture shows the high impact of pitting corrosion on the safety of RC beams.

Based on these results, the effect of the surrounding environment conditions that help in the growth and development of corrosion forms, as well as the applied external loads, have the greatest impact on safety levels for reinforced concrete beams. This research concluded that more advanced approaches should be used to redevelop the LSF variables, especially the ones based on empirical correlations and fitting approaches such as the corrosion growth parameters. The influence of the external loads on corrosion diffusion should be examined in future works, which will aid in describing corrosion phenomena with more real-world conditions. Among the suggested approaches is the use of artificial intelligence methods and optimization techniques.

## Figures and Tables

**Table 1:** Descriptive statistical properties of the basic random variables of the reinforced concrete beam.

Variable	Description	Mean	CoV	Distribution
$f_{c}$	Concrete compressive resistance (MPa)	30	0.18	Normal
$f_{y}$	Steel yield tension (MPa)	400	0.11	Log normal
*D*_0_	Bar diameter (mm)	18	0.05	Normal
*M* *_n_*	Applied moment (kN-m)	120	0.12	Gumbel
*b*	Section width (mm)	350	0.07	Normal
*d*	Effective height (mm)	500	0.07	Log normal
*C*	concrete cover (mm)	50	0.12	Normal
*η*	Model coefficient	1	0.1	Normal
α	Yield empirical coefficient	0.005	0.12	Log normal
*K*	Resistance ratio	0.6	0.05	Normal
λ	Load coefficient	1.05	0.1	Normal
*R*	The maximum to the average corrosion ratio	6	0.2	Gumbel
*T*	Corrosion initiation time (years)	variable	0.35	Log normal

## Data Availability

The data presented in this study are available on request from the corresponding author.
